# Wound-Healing Peptides for Treatment of Chronic Diabetic Foot Ulcers and Other Infected Skin Injuries

**DOI:** 10.3390/molecules22101743

**Published:** 2017-10-18

**Authors:** Ana Gomes, Cátia Teixeira, Ricardo Ferraz, Cristina Prudêncio, Paula Gomes

**Affiliations:** 1 LAQV-REQUIMTE, Departamento de Química e Bioquímica, Faculdade de Ciências, Universidade do Porto, Rua do Campo Alegre 687, P-4169-007 Porto, Portugal; anasmgomes@gmail.com (A.G.); ricardoferraz@eu.ipp.pt (R.F.); 2 Ciências Químicas e das Biomoléculas, Escola Superior de Saúde–Instituto Politécnico do Porto, Rua Dr. António Bernardino de Almeida 400, P-4200-072 Porto, Portugal; cps@estsp.ipp.pt; 3 i3S–Instituto de Investigação e Inovação em Saúde, Universidade do Porto, Rua Alfredo Allen 208, P-4200-135 Porto, Portugal

**Keywords:** antimicrobial, chronic infection, diabetes, peptides, ulcers, wound-healing, skin and soft tissue infections (SSTI)

## Abstract

As the incidence of diabetes continues to increase in the western world, the prevalence of chronic wounds related to this condition continues to be a major focus of wound care research. Additionally, over 50% of chronic wounds exhibit signs and symptoms that are consistent with localized bacterial biofilms underlying severe infections that contribute to tissue destruction, delayed wound-healing and other serious complications. Most current biomedical approaches for advanced wound care aim at providing antimicrobial protection to the open wound together with a matrix scaffold (often collagen-based) to boost reestablishment of the skin tissue. Therefore, the present review is focused on the efforts that have been made over the past years to find peptides possessing wound-healing properties, towards the development of new and effective wound care treatments for diabetic foot ulcers and other skin and soft tissue infections.

## 1. Introduction

In 2015, 8.8% of the adult population worldwide is estimated to have diabetes. This figure is projected to rise to 10.4% by the year 2040, which underpins an alarming public health problem that needs urgent control [1]. Diabetes is a chronic disease that stems from pancreas dysfunction, impairing normal production of insulin which leads to high, and also fluctuating, blood glucose levels. This, in turn, leads to an imbalance in the homeostatic regulation of the body, causing serious health problems such as blindness, kidney failure, heart disease, venous insufficiency and peripheral neuropathy [1]. A combination of these latter two conditions with improper footwear leads to loss of sensitivity in the feet of diabetic patients, which facilitates installment of chronically-infected lesions, the so-called diabetic foot ulcers (DFU), ultimately leading to limb amputations [1,2]. According to a 2012 report from the European Wound Management Agency, about 2% of the population in developed countries suffers from non-healing injuries like DFU, and it is estimated that 25–50% of hospital beds are occupied by patients with such an acute wound. DFU are the most common reason for hospitalization of people with diabetes, and about one in every six people with diabetes develops at least one foot ulcer during their lifetime. Moreover, 40–70% of all lower extremity amputations are related to diabetes, of which 85% are preceded by DFU [3]. In addition, the quite recent “EpiCast report: Diabetic Foot Ulcers-Epidemiology Forecast to 2025”—based on data from seven countries, namely US, France, Germany, Italy, Spain, UK and Japan—points to over 1 million cases in 2015 and an estimated increase rate of 4.03% per year, which will expectedly lead to approximately 1.5 million cases by 2025 [4].

**Figure 1:**
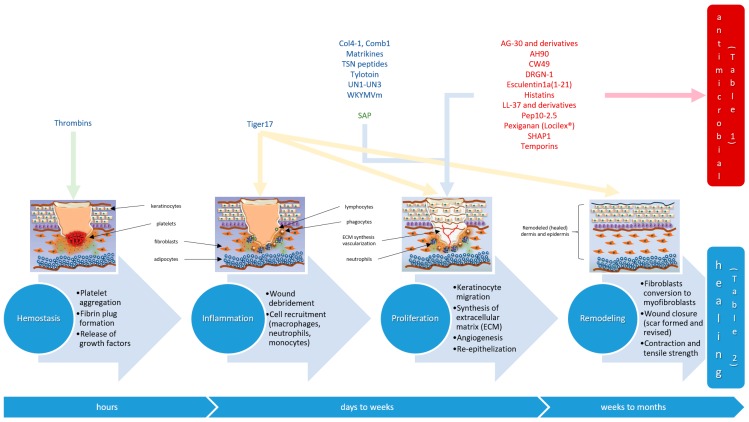
Schematic overview of major stages of wound-healing. Most wound-healing peptides were found to act on one or more biochemical pathways of the proliferation stage, although a few have been reported to act either on earlier stages (e.g., thrombin-related peptides) or on multiple stages (e.g., Tiger17)–see text. Examples of wound-healing antimicrobial peptides (Table 1) in dark red, and of wound-healing non-antimicrobial peptides (Table 2) in dark blue; SAP: self-assembling peptides (green).

Management of DFU should always start with prevention, through policies for foot inspection and use of custom therapeutic footwear to prevent ulceration, as DFU are tremendously difficult to heal once installed [3,5]. The wound-healing process comprises complex biological mechanisms triggered by the injury, involving four key steps: (i) hemostasis; (ii) inflammation; (iii) proliferation and (iv) tissue remodeling (Figure 1). In each of these processes, several cellular and biophysical events occur, such as monocyte differentiation to macrophage, neutrophil infiltration and lymphocyte infiltration, in the inflammation stage; or increased angiogenesis and re-epithelialization by fibroblast migration, during the proliferation phase. These events may be impaired by different factors, which can be divided into local and systemic ones. As a local factor, we can take the example of wound oxygenation, which influences healing. In what concerns systemic factors, we can take the example of a diabetic patient and respective individual characteristics: age, nutrition or other diseases (e.g., fibrosis, uremia), which may give rise to problems that impair healing, such as hypoxia, dysfunction in fibroblasts and epidermal cells, or incorrect angiogenesis, among others [6].

Management of DFU requires good knowledge of their complexity for a correct classification of stage and severity; for instance, it is important to distinguish sloughy and necrotic tissues (severe infection) from granulation and epithelializing ones (healing wound). Treatment of severe DFU usually requires initial debridement to remove sloughy and necrotic tissue, followed by the use of scaffolds to support skin rebuilding, along with efficient antimicrobial protection through a combination of several topical agents [7,8]. Collagen-based wound dressings have been used as healing scaffolds in both skin burns and DFU with overall promising results [9]. Those are based on animal collagens (bovine, porcine, equine), acting as biocompatible wound/environment barriers, rather than replacing or promoting formation of endogenous (human) collagen. While efficient production of either recombinant human collagen or “perfect” collagen-like peptides remains an unmet goal [10], an alternative sensible option may be the design of formulations comprising collagen-boosting instead of collagen-like components. Actually, collagen-boosting peptides, e.g., Matrikines^®^ like KTTKS, are already used in cosmetics to promote extracellular matrix (ECM) production, rebuilding structure and restoring all functions of healthy skin [11,12]. Moreover, many antimicrobial peptides (AMP) can also act as wound-healing peptides (WHP), thus displaying the dual antimicrobial and tissue-regenerating properties highly desired in novel topical formulations for treatment of DFU [13,14].

Today, the therapeutic value of AMP against infectious pathogens, including antibiotic-resistant strains, is well recognized [15,16], and topical formulations of AMP have reached either the market, like polmyxins or gramicidins, or clinical trials, such as Pexiganan (Locilex^®^), Lytixar, OP-145 or Novexatin, for cutaneous, ocular and otorhinolaryngological infections [17,18]. Understandably, the literature is quite rich in both original reports and comprehensive reviews on AMP of either natural or synthetic origin, addressing all relevant aspects in the topic, from discovery or design to spectrum of activity, mechanisms of action, patents, and clinical applications [15,16,17,18,19,20,21,22]. In contrast, literature focusing on the wound-healing properties exhibited by some peptides is still emergent. As such, and considering the undeniable timeliness of the topic, this review is focused on peptides whose wound-healing effects may find application in the topical treatment of DFU and other skin and soft tissue infections (SSTI).

## 2. Wound-Healing Antimicrobial Peptides

Commonly, non-healing wounds are simultaneously colonized by different bacteria; bacterial species most frequently isolated from such wounds typically include *Staphylococcus aureus* (93.5%), *Enterococcus faecalis* (71.1%) and *Pseudomonas aeruginosa* (52.2%), among others [23]. In healthy individuals, the innate immune system produces gene-encoded AMP that control proliferation of pathogenic microorganisms on skin, while triggering a signaling cascade in response to injuries [24]. However, due to their typical venous insufficiency and other diabetes-related conditions, diabetic patients are not immunocompetent, which favors formation of bacterial biofilms that are highly refractive to current antibiotics [25]. Topical application of AMP on the infected injury may overcome this barrier, as AMP have shown the ability to: (i) prevent infection due to their antimicrobial activity; (ii) reduce the pro-inflammatory response; (iii) promote cell migration and proliferation [14]. As such, many known AMP have been reported to also display wound-healing properties; some of which are addressed in this section and compiled in Table 1.

### 2.1. Human-Based Defence Peptides

Human genetically-encoded AMP that are expressed on skin, among other tissues, as part of the body’s immune response to injury include the well-known human β-defensins (hBDs) [31], cathelicidin LL-37 [35] and dermcidins [42]. One of the endogenous AMP that gained more attention in the context of DFU healing was human cathelicidin LL-37. In 2008, Carretero et al. reported that in vivo adenoviral transfer of LL-37 AMP to excisional wounds in diabetic-ob/ob mice (mutant obese mice used as animal models of type II diabetes), improved re-epithelialization and granulation tissue formation [43]. This was later confirmed by Ramos et al., who tested both LL-37 and PLL-37 (LL-37 derivative with an N-terminal proline) in vitro and in vivo skin lesions with impaired wound-healing, as they found both peptides to promote re-epithelialization and angiogenesis [38]. Shortly after, in 2012, LL-37 was compared to another AMP, the innate defense regulator peptide 1018 (IDR-1018) [34]. The study revealed that IDR-1018 was less toxic than LL-37 in vitro, and demonstrated significantly accelerated wound-healing in *S. aureus*-infected porcine and non-diabetic murine wounds. However, the study showed that IDR-1018 neither accelerated healing nor differed significantly from LL-37 with regards to bacterial colonization of DFU in diabetic mice. Moreover, diabetes seemed to have a negative impact on mice immune responses, eventually blocking signaling pathways where innate defense regulators like LL-37 and IDR-1018 are involved [34].

Also in 2012, Rivas-Santiago et al. used primary cell cultures from DFU to study the expression and role of human endogenous AMP, including LL-37 and β-defensins hBD-1, hBD-2, hBD-3 and hBD-4, in wound-healing [32]. These authors found that although β-defensins are expressed in DFU, their production seems to be not high enough to contain infection and promote proper wound-healing. The study also showed that when compared with healthy skin, DFU biopsies presented low or null levels of LL-37, which might contribute to DFU pathogenesis. Moreover, topically-applied exogenous LL-37, i.e., peptide added to the wound site, was degraded by proteases presented in the wound [32]. These observations were later confirmed by McCrudden et al., who found that topically-administered LL-37 was unstable in the microenvironment of DFU, probably by the action of both host and bacterial proteinases that cleaved LL-37 into smaller peptides [44]. The authors thus suggested that to overcome the degradation process, LL-37 analogues should be developed to include some simple structural modifications, e.g., the replacement of α- by β-amino acids [44]. While investigating how to increase concentration of endogenous LL-37 at wound sites, Gonzalez-Curiel et al. found that the addition of 1,25-dihydroxyvitamin D_3_ and L-isoleucine to the wound increased production of hBD-2 and LL-37 during the regeneration process in primary cultures of cells from DFU sites [45]. All these promising findings on the wound-healing potential of LL-37 have culminated with this peptide entering clinical trials for venous leg ulcer treatment in 2014; results thereof suggested that LL-37 is safe and has a significant dose-response activity, although in no case could total wound closure be observed, which was attributed to the short trial period [46]. More recently, Song et al. immobilized an LL-37 derivative, Cys-KR12, onto electrospun silk fibroin (SF) nanofiber membranes [29]. Cys-KR12 includes residues 18–29 of the LL-37 sequence, and was chosen due to its antimicrobial and anti-biofilm action against four different bacterial strains (*S. aureus*, *S. epidermidis*, *E. coli* and *P. aeruginosa*). The peptide-modified membranes were found to promote proliferation of keratinocytes, fibroblasts and monocytes, all important players in the wound-healing process [29]. Taken together, results gathered thus far on the potential of LL-37 towards the healing of DFU and other SSTI are quite promising, regarding the use of LL-37 both for adequate formulation as a topical antimicrobial agent and for the coating of biomedical materials amenable to being applied as wound dressings.

At the same time as LL-37 was gaining more attention in the context of DFU healing, Nishikawa and co-workers developed an antimicrobial helical peptide—AG-30 (Table 1)—with angiogenic properties potentially similar to those of LL-37 [47]. However, since peptides are typically unstable in vivo, they engaged in the synthesis of AG30-related peptides as leads for clinical applications [26]. In this context, and in order to stabilize the AG-30 helical structure, the design strategy consisted of replacing several neutral residues (specifically proline, asparagine and serine) with cationic or hydrophobic amino acids [26]. As a result, AG30/5C (Table 1), where five residues of the original sequence AG30 were replaced by cationic amino acids, displayed improved antimicrobial and angiogenic activities when tested in vivo in a diabetic mouse wound-healing model with methicillin-resistant *S. aureus* (MRSA) infection [26]. By further evaluating the metabolic stability of AG30/5C, Tomioka et al. found that SR-0007 (Table 1), a 20-residue metabolite, stimulated cell proliferation at levels similar to those of the parent peptide [40]. However, as SR-0007 was rapidly degraded by human serum at 37 °C, a native L-lysine residue was replaced by its *D* enantiomer, yielding an analogue (SR-0379, Table 1) with significantly improved stability [40]. This peptide displayed antimicrobial activity against several distinct bacteria, including drug-resistant strains, and significantly accelerated wound-healing on a full-thickness wound model with a skin flap in a streptozotocin-induced diabetic rat model, holding great promise in the treatment of MRSA-positive diabetic ulcers [40].

Motivated by the fact that wounds in the oral cavity heal much faster than skin lesions, Oudhoff and co-workers engaged in a study to assess the factors in human saliva that contribute to its wound-healing properties [33]. By testing saliva and saliva protein fractions in an in vitro model for wound closure using an epithelial cell line, these researchers identified histatin-1 (Hst1), histatin-2 (Hst2) and histatin-3 (Hst3) (Table 1), rather than epidermal growth factor as primarily thought, as the major wound-healing factors in human saliva [33]. This finding was quite surprising because salivary histatins had, up to then, only been implicated in the antifungal saliva weaponry. Still, Oudhoff et al. found that the two activities—cell-stimulating and antimicrobial—are very distinct in their mode of action as the latter is independent of the chirality of the peptide and occurs via disruption of the phospholipid membrane of the target cell [33]. On the contrary, the stimulating activity on host cells involves a stereospecific interaction with a putative membrane receptor [33]. To ascertain whether histatins have potential applicability as general wound-healing agents, Oudhoff et al. further tested those peptides in a tissue-engineered epidermal skin that closely resembled native healthy skin [48]; the study showed that histatins enhance re-epithelialization by stimulating cell spreading and migration, but do not increase cell proliferation [48]. Additionally, by comparing the wound-healing activities of Hst1 fragments that differ by 2-residue-stepwise truncation in the *N*- or *C*-terminus, or both, they found that the region comprising residues 20 to 32 (SHREFPFYGDYGS) is critical for peptide biological activity [48]. Interestingly, they have also shown that constraining the conformation of Hst1 by cyclization resulted in a 1000-fold increase of its wound-healing activity in the in vitro wound-scratch assay [48]. These results have clearly established synthetic histatins as potential candidates for novel topical medicines to treat skin lesions, namely chronic ones. However, peptide-based therapeutics for wound-healing stimulation are sensitive to several proteases present in the chronic wound bed. Therefore, Boink et al. tested the in vitro stability of some histatin variants (Hst1, Hst2, cyclic Hst1 and minimal active domain of Hst1) in diluted chronic wound extracts, in order to determine if those peptides would remain stable in chronic ulcers long enough to exert their migration-stimulating activity [49]. The results showed a breakdown of ca. 50% for Hst1 and Hst2, and of ca. 20% for the minimal active domain of Hst1 and cyclic Hst1, in 8 h. As such, Boink et al. suggested that further investigations on the potential of the latter two Hst1 derivatives as topical therapeutics for SSTI should be undertaken [49].

### 2.2. Amphibian-Based Defence Peptides

Pexiganan, also known as MSI-78, is another AMP whose therapeutic potential against DFU has been widely recognized; it is a 22-residue linear cationic AMP analogue to magainins, which are natural AMP isolated from the skin of the African clawed frog *Xenopus laevis* [50]. By the turn of the century, 1% pexiganan acetate cream was proposed for topical treatment of mild to moderate DFU, as an alternative to the classical ofloxacin-based oral antibiotherapy [36]. Indeed, in the course of earlier clinical trials where patients were treated either with pexiganan cream or with active oral antibiotics, and compared with the placebo group, pexiganan’s performance proved it to be a possible alternative to oral antibiotics [51]. These findings triggered several research works focused on pexiganan and related peptides [52,53,54], and motivated the patenting of a 0.8% pexiganan acetate cream (Locilex^®^, earlier known as Cytolex) in several countries, by the pharmaceutical company Dipexium Pharmaceuticals, Inc. (Houston, TX, USA) in 2016 [55,56]. However, Locilex^®^ failed approval by the competent international organizations, on the grounds that its efficacy was not proven superior to that of classic oral antibiotics in any of the clinical trials undertaken to date, which include a recent Phase 3 clinical trial promoted by Dipexium, now merged with PLx Pharma, Inc. (Houston, TX, USA) [57]. Remarkably, AMP such as magainins and related peptides, like pexiganan, are not the only wound-healing peptides found, or inspired, in secretions from the skin of amphibians including frogs, toads and salamanders [58,59]. In fact, many known AMP are of amphibian origin, and most of them have revealed wound-healing properties, possibly contributing to the fast wound re-epithelialization typical in many species of the *Amphibia* class: e.g., the process takes less than 10 h in salamanders, while needing two to three days in mammalians. In agreement with this assumption, Liu et al. were able to isolate a new short (11 residues) AMP from the skin of *Odorrana grahami* frogs, CW49 (Table 1), which was found to promote angiogenesis while preventing excessive anti-inflammatory response when tested in DFU [14,28]. Interestingly, the same authors isolated another wound-healing AMP, peptide AH90 (Table 1), from the skin of the same frog species, and found it to have wound-healing activity, although this was not tested in DFU [27].

In addition to the above, there is a large collection of AMP of diverse origin whose healing ability has been demonstrated on SSTI other than DFU. Regardless of having been tested in DFU or not, any wound-healing AMP encloses the potential to become a useful topical agent against this particular type of SSTI. In this connection, one such recent example is the work performed by Di Grazia and co-workers, who have reported that temporins A and B (Table 1), two short AMP isolated from the skin of the European red frog *Rana temporaria*, promote in vitro wound-healing in a monolayer of immortalized human keratinocytes (HaCaT cells) [41]. Moreover, they have also found that both temporins, although to different extents, can reduce the number of *S. aureus* bacteria inside HaCaT keratinocytes in a dose-dependent manner. Temporin B, the most active of both peptides, kills ca. 80% of the bacterial cells at its highest non-cytotoxic dosage (16 μM) within 2 h [41]. These results suggested that temporins are attractive candidates for the generation of new therapeutics to treat *S. aureus*-related SSTI. The same researchers further reported that esculentin-1a(1-21) (Table 1), a derivative of AMP esculentin-1a isolated from the skin of the frog *Rana esculenta*, significantly stimulates migration of HaCat cells over a wide range of peptide concentrations (0.025–4 μM), being notably more efficient than LL-37 [30]. This observation, allied with the established antimicrobial activity for the same esculentin-1a derivative, namely its high anti-*Pseudomonal* activity, makes this AMP a promising wound-healing promoter, especially against chronic, often *Pseudomonas*-infected, skin ulcers [30].

### 2.3. Synthetic Peptides

Synthetic AMP, designed for improved structural stability and high antimicrobial activity under physiological conditions, have been found to also present wound-healing activity [37,39]. One example was reported in 2014 by Kim et al., who demonstrated that SHAP1 (Table 1), a synthetic AMP with potent antimicrobial activity against fungi and bacteria in the presence of 200 mM NaCl, also produces wound closure both in vitro and in vivo [39]. Indeed, SHAP1 displayed stronger in vitro wound-healing activity than LL-37 by inducing HaCaT cell migration, which was reported to occur via transactivation of the epidermal growth factor receptor [39]. One important finding was that topical application of SHAP1 accelerated closure and healing of full-thickness excisional wounds in mice at the same low concentration (1 μM) that was used in in vitro assays [39], indicating that SHAP1 stands as a highly promising topical agent for treatment of SSTI.

In 2016, Pfalzgraff et al. also assessed the potential of Pep19-2.5 (Table 1), a synthetic anti-lipopolysaccharide peptide presenting antimicrobial activity for Gram-negative and Gram-positive bacteria, for therapeutic applications in bacterial skin infections [37]. The results showed that Pep19-2.5 displays low in vitro cytotoxicity in primary human keratinocytes and fibroblasts. Furthermore, it reduces the immune and inflammatory responses in skin cells stimulated with potent bacterial pathogenicity factors, with concurrent potent stimulation of keratinocyte migration; this suggests that Pep19-2.5 might be a promising option for the treatment of acute and chronic wounds most commonly infected with *S. aureus* and *P. aeruginosa* [37].

An interesting and very recent example of a highly promising synthetic wound-healing AMP is that of DRGN-1, developed by Chung et al. who sought inspiration in a histone H1-derived peptide from the Komodo dragon (*Varanus komodoensis*) [60]. DRGN-1 was found to display potent antimicrobial and anti-biofilm activity, and to permeabilise bacterial membranes, while being equally able to promote significant tissue regeneration ability in vitro; according to the authors, based on data from scratch wound closure assays, the wound-healing ability of DRGN-1 is due to promotion of migration of HEKa keratinocytes and activation of the EGFRSTAT1/3 pathway. The potential of DRGN-1 as a wound-healing agent was further confirmed in vivo, as the peptide was able to significantly enhance wound-healing in both uninfected and mixed biofilm (*P. aeruginosa* and *S. aureus*)-infected murine wounds [60].

## 3. Tissue Regeneration Peptides

As seen in the previous section, some AMP are considered promising agents for wound care because, in addition to their intrinsic antimicrobial activity, they can influence different mechanisms of the wound-healing orchestration such as inflammation, epithelialization, tissue granulation and remodeling. Still, although some wound-healing peptides do not display antimicrobial properties, some of them have great therapeutic potential towards healing chronically-infected wounds, like DFU and other SSTI. Recent examples of such peptides are summarized in Table 2 and next reviewed.

### 3.1. Human-Based Peptides

One class of non-antimicrobial peptides that promote tissue repair is that of thrombins. Thrombins and related peptides are known for their important role in blood clotting, whereby they promote cleavage of soluble fibrinogen into fibrins. TP-508 (Table 2) is a peptide fragment of the receptor-binding domain of native human thrombin, whose saline solutions, also known as Chrysalin^®^, have been tested for DFU healing. In the course of phase I and phase II clinical trials, Chrysalin^®^ showed 45–72% of complete healing compared with the placebo group, without relevant local wound reactions or observable adverse effects [66].

In 2011, Demidova-Rice et al. reported that several bioactive peptides, liberated from capillary endothelial cells degradation by *Clostridium histolyticum* collagenase, facilitate endothelial responses to injury and angiogenesis [63]. Two peptides in particular, Col4-1 and Comb1 (Table 2), when used at 10–100 nM, were found to increase rates of microvascular endothelial cell proliferation by up to 47% and in vitro angiogenesis by 200% when compared with serum-stimulated controls [63]. These researchers later assessed whether these peptides could improve wound-healing in vivo, and found that Comb1, when applied into impaired cranial dermal wounds created in cyclophosphamide-treated mice, could stimulate the rate of wound closure [68]. Moreover, they identified three novel peptides derived from human platelet-rich plasma, UN1-UN3 (Table 2), which stimulate epithelial migration as well as angiogenesis in vitro [68]. Yet, UN3 was shown as the most potent stimulator of cellular responses, by causing a 250% increase in angiogenic response, a 50% increase in endothelial proliferation and a tripling of epithelial cell migration in response to injury [68]. Additionally, results of in vivo experiments where UN3 and Comb1 were added together to impaired cranial wounds in cyclophosphamide-treated mice showed an increase of the number of blood vessels present in the wound beds. Interestingly, combining the peptides before addition to the cells in vitro did not produce any synergistic effects [68]. Motivated by these promising findings, the same authors further investigated whether Comb1 and UN3 could stimulate healing in a diabetic porcine model highly reminiscent of human healing impairments in type-I and -II diabetes [70]. This study revealed that both peptides significantly improve angiogenesis and re-epithelialization in diabetic porcine wounds when compared to saline-treated controls. Although the authors found that both peptides stimulate wound closure through upregulation of cytokines and multiple reparative growth factors, their precise mechanism of action remains unclear [70]. At the same time, this same research group postulated that Santyl^®^, a FDA approved debridement agent containing *Clostridial* collagenases and other non-specific proteases, could also provide healing through the production of bioactive peptides able to stimulate tissue and cellular responses to injury [67]. Indeed, they were able to identify several bioactive peptides (TSN1-8, TSN11-12, TSN14-17; Table 2) when doses of Santyl^®^ collagenase, comparable to those used clinically, were applied to human dermally-derived capillary ECM [67]. Interestingly, there is little overlap between these peptides, arising from Santyl^®^ collagenase, and the endothelial peptides (Comb1 and UN3) produced by purified *Clostridial* collagenase. The authors next found that the individual peptides released from Santyl^®^-digested ECM, as well as the combinatorial peptides (TSN9-10, TSN13 and TSN18; Table 2) induced wound-healing in vitro, highlighting TSN6 and TSN18 as those having the greatest stimulatory activity over all cell types and parameters assayed [67]. When they further applied these peptides to healing-impaired cyclophosphamide-treated mice, they observed that 0.1 mg/mL TSN18 stimulated a >100% increase of epithelial responses, while 1.0 mg/mL TSN18 produced a 60% increase in epithelialization when compared to controls [67]. Overall, these results indicate TSN18 as an exciting new opportunity for creating advanced wound-healing therapies. In parallel to the aforementioned studies, the therapeutic effect of WKYMVm (Table 2)—a short synthetic agonist of formyl peptide receptor 2 known to promote angiogenesis—on DFU was investigated by Kwon and co-workers [69]. As such, this peptide was tested in cutaneous wounds of streptozotocin-induced diabetic rats, where it was found to accelerate angiogenesis, granulation tissue formation, re-epithelialization and wound closure, suggesting its potential usefulness to treat chronic cutaneous wounds [69].

**Figure 2:**
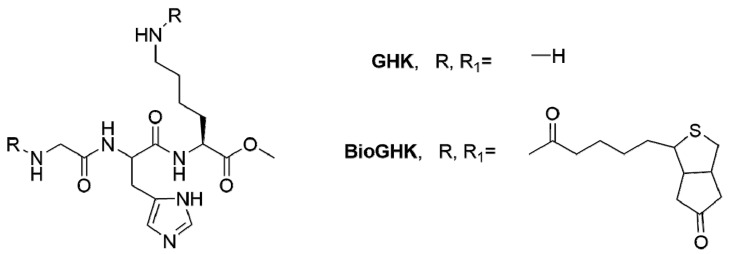
Structure of the matrikine tripeptide GHK and its biotinylated derivative BioGHK [62,72].

Findings like the above motivated other approaches to non-antimicrobial wound-healing peptides that sought inspiration on platelet-rich plasma and ECM proteins. This gave rise to new classes of wound-healing peptides, such as the so-called matrikines, matricryptins, and laminin analogues, among others. Matrikines, i.e., ECM-inspired peptides, have been described to promote collagen formation in fibroblasts, leading to skin repair, but their therapeutic potential has also been surveyed for cancer, inflammation and neurodegenerative disorders, as well as for cosmetic applications [12,71]. A typical matrikine is tripeptide GHK (Figure 2), well known for its ability to form copper(II) complexes, anti-inflammatory and wound-healing properties, as well as for its capacity to improve skin density and firmness [62]. In 2005, Arul et al. reported that a biotinylated GHK derivative (BioGHK, Figure 2) incorporated in a collagen matrix showed better wound-healing in vivo than the collagen matrix alone [72].

Later on, the same group tested the same system in skin wounds of diabetic rats, and were able to observe an increase in collagen biosynthesis, along with activation and growth of fibroblasts [73]. This result is rather encouraging regarding the potential of matrikines for topical treatment of DFU and other SSTI. A very recent example of the tremendous potential of matrikines regards the work from Banerjee and co-workers, who have investigated the wound-healing ability of E1 (Table 2), a cryptic peptide derived from bovine Achilles tendon collagen [64]. The results showed that topical administration of E1 at a concentration of 60 μM greatly increased wound closure throughout the duration of the experiment, while >90% tissue restoration was observed on the 12^th^ day, when compared to 55% restoration for the control group [64]. Similarly, Kwon and co-workers have explored the effects of Ac-PGP (Table 2), a peptide derived from degradation of collagen, on cutaneous wound-healing and neovascularization in human endothelial progenitor cells (hEPCs) [61]: following enhanced proliferation, migration and tube-forming activity of hEPCs upon in vitro treatment with Ac-PGP, in vivo assays further demonstrated that topical application of this peptide accelerates wound closure by 11 to 23% with promotion of neovascularization, in comparison with the control group [61]. These results suggested that Ac-PGP should be pursued as a potential therapeutic product for treatment of chronic and acute wounds.

### 3.2. Amphibian-Based Peptides

As mentioned in the previous section, several AMP of diverse origin, namely those isolated from amphibians, have demonstrated healing properties on different wound types in addition to their intrinsic antimicrobial activity. Interestingly, some peptides derived from such AMP were found to lose their antimicrobial ability, while retaining strong wound-healing activity. One such example is that of tylotoin (Table 2), a short peptide derived from the *C*-terminus of a cathelicidin extracted from the skin of the *Tylototriton verrucosus* salamander [59]. Mu et al. found that, although tylotoin did not show any antimicrobial activity, it significantly improved in vitro proliferation and migration of vascular endothelial cells, fibroblasts and keratinocytes [59]. In vivo assays further demonstrated the wound-healing ability of tylotoin as its topical application markedly enhanced wound-healing in a mouse model of full-thickness skin wounding. The same work further revealed that tylotoin also promotes the release of transforming growth factor β1 and interleukin 6, which are essential in the wound-healing process, thus suggesting tylotoin as a valuable template for development of novel wound-healing agents [59]. Similarly, Tang and co-workers have reported Tiger17 (Table 2), a cyclic peptide derived from the AMP tigerinin isolated from skin secretions of the *Fejervarya cancrivora* frog, as a non-AMP with excellent skin wound-healing ability [65]. These authors found that topical application of Tiger17 greatly diminished wound closure time in a full-thickness skin wound mouse model, and that the peptide was involved in three stages of wound-healing: (i) the inflammatory stage, by recruiting macrophages to the wound site; (ii) re-epithelialization and granulation tissue formation, by promoting proliferation and migration of both keratinocytes and fibroblasts; and (iii) the tissue remodeling phase, by activating mitogen-activated protein kinases signaling pathways and by stimulating the release of interleukin 6 and transforming growth factor β1 in murine macrophages [65]. Altogether, these results clearly indicate Tiger17 as a promising wound-healing lead.

### 3.3. Self-Assembling Peptides

**Figure 3:**
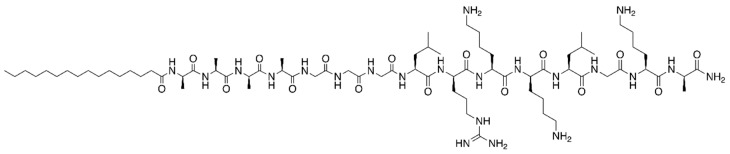
Structure of the peptide amphiphile PA, developed by Ranjagam and co-workers [75].

One other quite promising class of peptides for wound-healing, given their ability to promote tissue regeneration, is that of self-assembling peptides (SAP). Peptide amphiphiles (hybrid compounds that comprise both an alkyl and a peptide tail) are examples of important SAP-based scaffolds for tissue engineering [74]. By virtue of their properties, SAP and other peptides able to promote tissue regeneration can assume an important role in wound-healing, being potentially useful for topical treatment of DFU and other SSTI. In 2006, Ranjagam et al. developed peptide amphiphile PA (Figure 3) [75], based on the consensus sequence XBBBXXBX (X = hydrophobic amino acid; B = basic amino acid) earlier designed for heparin-binding [69], since heparin plays an important role in nanostructure nucleation into macromolecules able to efficiently activate, and bind to, angiogenic growth factors.

**Figure 4:**
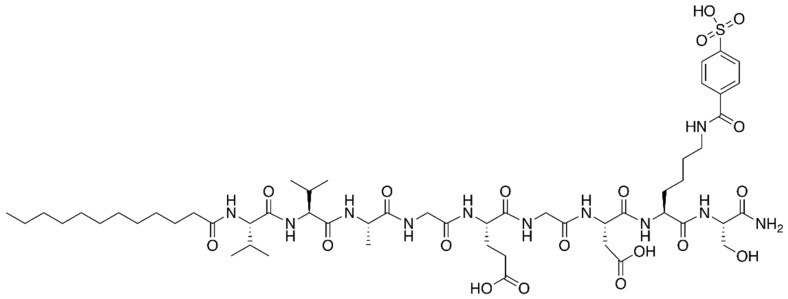
Structure of the heparin-mimetic peptide amphiphile developed by Mammadov and co-workers [76].

The authors observed that aqueous mixtures of PA, heparin, vascular endothelial, and fibroblast 2 growth factors, were able to promote extensive blood vessel formation in vivo. Moreover, they found a peptide sequence that self-assembled into nanofibers upon interaction with heparin [75]. Later, Mammadov et al. developed a heparin-mimetic PA (hmPA, Figure 4), designed to display the angiogenesis-promoting properties of heparin, without the need to add this glucosaminoglycan, or exogenous growth factors; their working hypothesis was confirmed, as hmPA was able, by itself, to promote angiogenesis and tissue regeneration [76].

These findings motivated Senturk et al. to test the ability of hmPA-based gels to heal diabetic wounds, in streptozotocin-induced diabetic mice; results confirmed the bioactive gels to have a key role in both inflammation and proliferation phases of wound-healing, by promoting angiogenesis, re-epithelialization and inflammatory response [77].

Several wound dressings are available in the clinics for superficial treatment of mild to severe wounds, starting from simple gauze up to highly complex materials which include films, hydrogels, foams, hydrofibers and engineered skin substitutes [78]. Current biomedical approaches for advanced wound care aim at providing scaffolds to support skin rebuilding, along with efficient antimicrobial protection, given that over 50% of chronic SSTI are associated with bacterial biofilms underlying severe infections that contribute to tissue destruction, delayed wound-healing, and other complications [8]. Under certain conditions, SAP self-assemble to produce supramolecular structures; hence, several groups have looked into their use as appropriate scaffolds for the production of wound dressings. This is a broad and fascinating topic that has been reviewed in great detail by others, and is therefore outside the scope of the present review [79,80]. Still, some recent examples of peptide-based wound dressings will be addressed next.

## 4. Peptide-Based Wound Dressings

Collagen-based wound dressings have been used as healing scaffolds in both skin burns and DFU with overall promising outcomes, as collagen plays an important role in tissue regeneration, stabilizing the vascular and cellular components in the wound [9,81]. Collagen applied in saline-moistened gauze has been tested in DFU, with encouraging results [82]. Recently, Choudhary et al. reported the wound-healing activity of collagen-based dressings as compared to conventional (dry) dressings; the study included 60 diabetic patients, most of them with traumatic foot injuries followed by installation of a diabetic ulcer, with those being treated with the collagen-based dressing showing more pronounced regression of infection and appearance of granulation tissue [83]. Still, collagen alone cannot provide antimicrobial protection, which motivated research initiatives where collagen was mixed with other biocompatible biopolymers, such as chitosan, whose intrinsic antimicrobial and wound-healing properties are very well known [84]. Other polysaccharides (e.g., dextrin and alginate) and AMP have also been considered as relevant components to build new generation wound dressings able to simultaneously provide (i) scaffold for skin rebuilding; (ii) tissue-regeneration; and (iii) antimicrobial action [85,86]. In this context, Xiao et al. recently prepared a chitosan-collagen hydrogel grafted with peptide QHREDGS, an angiopoietin-1 derivative that promotes migration of human primary keratinocytes and activates kinase B Akt, thus being potentially useful against DFU. The peptide-tethered hydrogel was proven to (i) protect keratinocytes against hydrogen peroxide stress in a dose-dependent manner; (ii) promote migration of keratinocytes from both healthy and diabetic people; (iii) accelerate and enhance wound closure; and (iv) promote angiogenesis, as the total number of blood vessels was greater in the peptide-hydrogel treated wounds, although no difference was observed in vessel density or size inside the wound [87].

Cellulose and derivatives embedding wound-healing peptides have also been tested on DFU. One recent example concerns the use of peptide ACT1 (RQPKIWFPNRRKPWKKRPRPDDLEI), a mimetic of the *C*-terminus of connexin43, a protein identified as potentially useful for treatment of DFU. ACT1 was embedded in a hydroxyethyl-cellulose hydrogel, and this formulation was topically applied on DFU [88]. As the results demonstrated the peptide-embedded gel to expedite re-epithelization while being non-toxic [88], a randomized phase III clinical trial was initiated in July 2015 to determine whether this gel (Granexin) is safe and effective in the treatment of diabetic foot ulcers [89].

Whole cells have also been regarded as potentially useful “biomaterials” for topical delivery of wound-healing peptides. Very recently, Seo et al. tested a combination of peptide exendin-4 (HGEGTFTSDLSKQMEEEAVRLFIEWLKNGGPSSGAPPPS), a glucagon-like peptide-1 receptor agonist, with adipose tissue-derived stem cells, for healing DFU on db/db mice, which are animal models of diabetic dyslipidemia. This combination performed significantly better than local injection of only the stem cells, but it remains to be determined whether the effect of the combination is synergistic or additive [90].

## 5. Conclusions

Wound-healing is a complex multi-stage process towards the rebuilding of skin. In many diverse pathologies, such as diabetes mellitus, normal wound-healing is impaired, which may lead to severe complications, from ulcers to chronic skin infections. As such, advanced biomedical approaches for effective wound care aim at providing antimicrobial protection to the open wound together with promotion of fast and correct healing, so that a fully functional healthy skin can be swiftly restored. Today, the therapeutic value of AMP against infectious pathogens, including parasites, is well recognized, and several topical formulations containing AMP are currently in clinical use. In parallel, a plethora of wound-healing peptides and peptide-grafted dressings has been unveiled in the past few years, holding great promise for novel strategies for treatment of chronically infected wounds. The development of peptide-based materials for topical application in DFU and other SSTI is still in its infancy, and its future seems quite promising and exciting: based on findings made thus far, future joint efforts of peptide scientists and healthcare professionals working in this area will likely produce innovative solutions to this health issue, increasingly prevalent worldwide.

## Figures and Tables

**Table 1:** Examples of peptides with dual antimicrobial and wound-healing properties. Peptide sequences are displayed using the one-letter amino acid code, as per the IUPAC-IUBMB Joint Commission on Biochemical Nomenclature rules.

Peptide Name	Peptide Sequence	Reference
AG30	MLSLIFLHRLKSMRKRLDRKLRLWHRKNYP	[26]
AG30/5C	MLKLIFLHRLKRMRKRLKRKLRLWHRKRYK	
AH90	ATAWDFGPHGLLPIRPIRIRPLCG	[27]
CW49	APFRMGICTTN	[28]
Cys-KR12	KRIVKRIKKWLR	[29]
Esculentin-1a(1-21)	GIFSKLAGKKIKNLLISGLKG	[30]
hBD-1	DHYNCVSSGGQCLYSACPIFTKIQGTCYRGKAKCCK (Disulfide bridges: 5-34, 12-27, 17-35)	[31,32]
hBD-2	GIGDPVTCLKSGAICHPVFCPRRYKQIGTCGLPGTKCCKKP (Disulfide bridges: 8-37, 15-30, 20-38)	
hBD-3	GIINTLQKYYCRVRGGRCAVLSCLPKEEQIGKCSTRGRKCCRRKK (Disulfide bridges: 11-40, 18-33, 23-41)	
hBD-4	ELDRICGYGTARCRKKCRSQEYRIGRCPNTYACCLRK (Disulfide bridges: 6-33; 13-27; 17-34)	
Histatin-1	DSHEKRHHGYRRKFHEKHHSHREFPFYGDYGSNYLYDN	[33]
Histatin-2	RKFHEKHHSHREFPFYGDYGSNYLYDN	
Histain-3	DSHAKRHHGYKRKFHEKHHSHRGYRSNYLYDN	
IDR-1018	VRLIVAVRIWRR	[34]
LL-37	LLGDFFRKSKEKIGKEFKRIVQRIKDFLRNLVPRTES	[35]
**MSI-78 (pexiganan)**	**GIGKFLKKAKKFGKAFVKILKK**	[36]
Pep19-2.5	GCKKYRRFRWKFKGKFWFWG	[37]
PLL-37	PLLGDFFRKSKEKIGKEFKRIVQRIKDFLRNLVPRTES	[38]
SHAP1	APKAMKLLKKLLKLQKKGI	[39]
SR-0007	MLKLIFLHRLKRMRKRLKRK	[40]
SR-0379	MLKLIFLHRLKRMRKRLkRK ^1^	
Temporin A	FLPLIGRVLSGIL	[41]
Temporin B	LLPIVGNLLKSLL	

^1^ Lowercase letters indicate D-amino acid residues; Peptide highlighted in bold was entered into clinical trials for its assessment in the treatment of diabetic foot ulcers; S: phosphoserine.

**Table 2:** Examples of non-antimicrobial peptides presenting wound-healing activity. Peptide sequences are displayed using the one-letter amino acid code, as per the IUPAC-IUBMB Joint Commission on Biochemical Nomenclature rules.

Peptide Name	Peptide Sequence	Reference
Ac-PGP	*N*-acetylated-PGP	[61]
BioGHK	Biotinylated-GHK	[62]
Col4-1	MFRKPIPSTVKA	[63]
Comb1	DINECEIGAPAGEETEVTVEGLEPG	
E1	GETGPAGPAGPIGPVGARGPAGPQGPRGDKGETGEQ	[64]
Tiger17	WCKPKPKPRCH	[65]
**TP-508**	**AGYKPDEGKRGDACEGDSGGPFV**	[66]
TSN1	NFQGVQNRFVFGTP	[67]
TSN2	MENAELDVPIQSVFTR	
TSN3	NTDNIYPESSC	
TSN4	PYLGYVFK	
TSN5	MQTVAQLFKTVSSLSLST	
TSN6	HSPDIQLQKGLTFEPIQIK	
TSN7	STITQPYKTLNNARSP	
TSN8	RPGPSPEGTGQSYNY	
TSN9	MENAELDPPYLGYVFK	
TSN10	TGQSYNQYSQRPYLGVYVFK	
TSN11	LYGQTPLETL	
TSN12	ELADSPALEIG	
TSN13	LYGQTPLETLELADSPALEIG	
TSN14	VSGNTVEYALPTLE	
TSN15	LDSPTAPTVQSTALTWRP	
TSN16	LDGSAPGPLYTGSALDF	
TSN17	GSEGVRSGRSG	
TSN18	QPQPLPSPGVGGKN	
Tylotoin	KCVRQNNKRVCK	[59]
UN1	ELLESYIDGR	[68]
UN2	TATSEYQTFFNPR	
UN3	ELLESYIDGRPTATSEYQTFFNPR	
WKYMVm	WKYMVm ^1^	[69]

^1^ Lowercase letters indicate D-amino acid residues; Peptide highlighted in bold was entered into clinical trials for its assessment in the treatment of diabetic foot ulcers.

## References

[1] International Diabetes Federation (2015). IDF Diabetes Atlas.

[2] Uckay I., Gariani K., Pataky Z., Lipsky B.A. (2014). Diabetic foot infections: State-of-the-art. Diabetes Obes. Metab..

[3] Alexiadou K., Doupis J. (2012). Management of diabetic foot ulcers. Diabetes Ther..

[4] Epicast Report: Diabetic Foot Ulcers-Epidemiology Forecast to 2025. https://www.researchandmarkets.com/research/l9r7nt/epicast_report.

[5] Hingorani A., LaMuraglia G.M., Henke P., Meissner M.H., Loretz L., Zinszer K.M., Driver V.R., Frykberg R., Carman T.L., Marston W. (2016). The management of diabetic foot: A clinical practice guideline by the society for vascular surgery in collaboration with the american podiatric medical association and the society for vascular medicine. J. Vasc. Surg..

[6] Guo S., Dipietro L.A. (2010). Factors affecting wound healing. J. Dent. Res..

[7] Kavitha K.V., Tiwari S., Purandare V.B., Khedkar S., Bhosale S.S., Unnikrishnan A.G. (2014). Choice of wound care in diabetic foot ulcer: A practical approach. World J. Diabetes.

[8] Murphy P.S., Evans G.R. (2012). Advances in wound healing: A review of current wound healing products. Plast. Surg. Int..

[9] Holmes C., Wrobel J.S., Maceachern M.P., Boles B.R. (2013). Collagen-based wound dressings for the treatment of diabetes-related foot ulcers: A systematic review. Diabetes Metab. Syndr. Obes..

[10] He L., Theato P. (2013). Collagen and collagen mimetic peptide conjugates in polymer science. Eur. Polym. J..

[11] Abu Samah N.H., Heard C.M. (2011). Topically applied kttks: A review. Int. J. Cosmet. Sci..

[12] Ricard-Blum S., Salza R. (2014). Matricryptins and matrikines: Biologically active fragments of the extracellular matrix. Exp. Dermatol..

[13] Hancock R.E., Haney E.F., Gill E.E. (2016). The immunology of host defence peptides: Beyond antimicrobial activity. Nat. Rev. Immunol..

[14] Mangoni M.L., McDermott A.M., Zasloff M. (2016). Antimicrobial peptides and wound healing: Biological and therapeutic considerations. Exp. Dermatol..

[15] Mahlapuu M., Hakansson J., Ringstad L., Bjorn C. (2016). Antimicrobial peptides: An emerging category of therapeutic agents. Front. Cell. Infect. Microbiol..

[16] Mai S., Mauger M.T., Niu L.N., Barnes J.B., Kao S., Bergeron B.E., Ling J.Q., Tay F.R. (2017). Potential applications of antimicrobial peptides and their mimics in combating caries and pulpal infections. Acta Biomater..

[17] Fox J.L. (2013). Antimicrobial peptides stage a comeback. Nat. Biotechnol..

[18] Fosgerau K., Hoffmann T. (2015). Peptide therapeutics: Current status and future directions. Drug Discov. Today.

[19] Kang H.K., Kim C., Seo C.H., Park Y. (2017). The therapeutic applications of antimicrobial peptides (AMPs): A patent review. J. Microbiol..

[20] Fuente-Nuñez C., Silva O.N., Lu T.K., Franco O.L. (2017). Antimicrobial peptides: Role in human disease and potential as immunotherapies. Pharmacol. Ther..

[21] Mishra B., Reiling S., Zarena D., Wang G. (2017). Host defense antimicrobial peptides as antibiotics: Design and application strategies. Curr. Opin. Chem. Biol..

[22] Travkova O., Moehwald H., Berezinski G. (2017). The interaction of antimicrobial peptides with membranes. Adv. Colloid Interface Sci..

[23] Suleman L. (2016). Extracellular bacterial proteases in chronic wounds: A potential therapeutic target?. Adv. Wound Care New Rochelle.

[24] Barton G.M. (2008). A calculated response: Control of inflammation by the innate immune system. J. Clin. Investig..

[25] Zhao G., Usui M.L., Lippman S.I., James G.A., Stewart P.S., Fleckman P., Olerud J.E. (2013). Biofilms and inflammation in chronic wounds. Adv. Wound Care.

[26] Nakagami H., Nishikawa T., Tamura N., Maeda A., Hibino H., Mochizuki M., Shimosato T., Moriya T., Morishita R., Tamai K. (2012). Modification of a novel angiogenic peptide, ag30, for the development of novel therapeutic agents. J. Cell. Mol. Med..

[27] Liu H., Mu L., Tang J., Shen C., Gao C., Rong M., Zhang Z., Liu J., Wu X., Yu H. (2014). A potential wound healing-promoting peptide from frog skin. Int. J. Biochem. Cell Biol..

[28] Liu H., Duan Z., Tang J., Lv Q., Rong M., Lai R. (2014). A short peptide from frog skin accelerates diabetic wound healing. FEBS J..

[29] Song D.W., Kim S.H., Kim H.H., Lee K.H., Ki C.S., Park Y.H. (2016). Multi-biofunction of antimicrobial peptide-immobilized silk fibroin nanofiber membrane: Implications for wound healing. Acta Biomater..

[30] Di Grazia A., Cappiello F., Imanishi A., Mastrofrancesco A., Picardo M., Paus R., Mangoni M.L. (2015). The frog skin-derived antimicrobial peptide esculentin-1a(1-21) NH2 promotes the migration of human hacat keratinocytes in an egf receptor-dependent manner: A novel promoter of human skin wound healing?. PLoS ONE.

[31] Dhople V., Krukemeyer A., Ramamoorthy A. (2006). The human beta-defensin-3, an antibacterial peptide with multiple biological functions. Biochim. Biophys. Acta.

[32] Rivas-Santiago B., Trujillo V., Montoya A., Gonzalez-Curiel I., Castaneda-Delgado J., Cardenas A., Rincon K., Hernandez M.L., Hernandez-Pando R. (2012). Expression of antimicrobial peptides in diabetic foot ulcer. J. Dermatol. Sci..

[33] Oudhoff M.J., Bolscher J.G., Nazmi K., Kalay H., van’t Hof W., Amerongen A.V., Veerman E.C. (2008). Histatins are the major wound-closure stimulating factors in human saliva as identified in a cell culture assay. FASEB J..

[34] Steinstraesser L., Hirsch T., Schulte M., Kueckelhaus M., Jacobsen F., Mersch E.A., Stricker I., Afacan N., Jenssen H., Hancock R.E. (2012). Innate defense regulator peptide 1018 in wound healing and wound infection. PLoS ONE.

[35] Duplantier A.J., van Hoek M.L. (2013). The human cathelicidin antimicrobial peptide ll-37 as a potential treatment for polymicrobial infected wounds. Front. Immunol..

[36] Lamb H.M., Wiseman L.R. (1998). Pexiganan acetate. Drugs.

[37] Pfalzgraff A., Heinbockel L., Su Q., Gutsmann T., Brandenburg K., Weindl G. (2016). Synthetic antimicrobial and lps-neutralising peptides suppress inflammatory and immune responses in skin cells and promote keratinocyte migration. Sci. Rep..

[38] Ramos R., Silva J.P., Rodrigues A.C., Costa R., Guardao L., Schmitt F., Soares R., Vilanova M., Domingues L., Gama M. (2011). Wound healing activity of the human antimicrobial peptide ll37. Peptides.

[39] Kim D.J., Lee Y.W., Park M.K., Shin J.R., Lim K.J., Cho J.H., Kim S.C. (2014). Efficacy of the designer antimicrobial peptide shap1 in wound healing and wound infection. Amino Acids.

[40] Tomioka H., Nakagami H., Tenma A., Saito Y., Kaga T., Kanamori T., Tamura N., Tomono K., Kaneda Y., Morishita R. (2014). Novel anti-microbial peptide SR-0379 accelerates wound healing via the PI3 kinase/Akt/mTOR pathway. PLoS ONE.

[41] Di Grazia A., Luca V., Segev-Zarko L.A., Shai Y., Mangoni M.L. (2014). Temporins a and b stimulate migration of hacat keratinocytes and kill intracellular *Staphylococcus aureus*. Antimicrob. Agents Chemother..

[42] Yamasaki K., Gallo R.L. (2008). Antimicrobial peptides in human skin disease. Eur. J. Dermatol..

[43] Carretero M., Escámez M.J., García M., Duarte B., Holguín A., Retamosa L., Jorcano J.L., Río M.D., Larcher F. (2008). In vitro and in vivo wound healing-promoting activities of human cathelicidin ll-37. J. Investig. Dermatol..

[44] McCrudden M.T.C., McLean D.T.F., Zhou M., Shaw J., Linden G.J., Irwin C.R., Lundy F.T. (2014). The host defence peptide ll-37 is susceptible to proteolytic degradation by wound fluid isolated from foot ulcers of diabetic patients. Int. J. Pept. Res. Ther..

[45] Gonzalez-Curiel I., Trujillo V., Montoya-Rosales A., Rincon K., Rivas-Calderon B., deHaro-Acosta J., Marin-Luevano P., Lozano-Lopez D., Enciso-Moreno J.A., Rivas-Santiago B. (2014). 1,25-dihydroxyvitamin D_3_ induces LL-37 and HBD-2 production in keratinocytes from diabetic foot ulcers promoting wound healing: An in vitro model. PLoS ONE.

[46] Gronberg A., Mahlapuu M., Stahle M., Whately-Smith C., Rollman O. (2014). Treatment with LL-37 is safe and effective in enhancing healing of hard-to-heal venous leg ulcers: A randomized, placebo-controlled clinical trial. Wound Repair Regen..

[47] Nishikawa T., Nakagami H., Maeda A., Morishita R., Miyazaki N., Ogawa T., Tabata Y., Kikuchi Y., Hayashi H., Tatsu Y. (2009). Development of a novel antimicrobial peptide, ag-30, with angiogenic properties. J. Cell. Mol. Med..

[48] Oudhoff M.J., Kroeze K.L., Nazmi K., van den Keijbus P.A., van’t Hof W., Fernandez-Borja M., Hordijk P.L., Gibbs S., Bolscher J.G., Veerman E.C. (2009). Structure-activity analysis of histatin, a potent wound healing peptide from human saliva: Cyclization of histatin potentiates molar activity 1000-fold. FASEB J..

[49] Boink M.A., Roffel S., Nazmi K., van Montfrans C., Bolscher J.G., Gefen A., Veerman E.C., Gibbs S. (2016). The influence of chronic wound extracts on inflammatory cytokine and histatin stability. PLoS ONE.

[50] Zasloff M. (1987). Magainins, a class of antimicrobial peptides from xenopus skin: Isolation, characterization of two active forms, and partial cdna sequence of a precursor. Proc. Natl. Acad. Sci. USA.

[51] Lipsky B.A., Holroyd K.J., Zasloff M. (2008). Topical versus systemic antimicrobial therapy for treating mildly infected diabetic foot ulcers: A randomized, controlled, double-blinded, multicenter trial of pexiganan cream. Clin. Infect. Dis.

[52] Gottler L.M., Ramamoorthy A. (2009). Structure, membrane orientation, mechanism, and function of pexiganan--a highly potent antimicrobial peptide designed from magainin. Biochim. Biophys. Acta.

[53] Monteiro C., Fernandes M., Pinheiro M., Maia S., Seabra C.L., Ferreira-da-Silva F., Costa F., Reis S., Gomes P., Martins M.C. (2015). Antimicrobial properties of membrane-active dodecapeptides derived from msi-78. Biochim. Biophys. Acta.

[54] Monteiro C., Pinheiro M., Fernandes M., Maia S., Seabra C.L., Ferreira-da-Silva F., Reis S., Gomes P., Martins M.C. (2015). A 17-mer membrane-active msi-78 derivative with improved selectivity toward bacterial cells. Mol. Pharm..

[55] Newswire P. Dipexium Pharmaceuticals Announces Issuance of Locilex^®^ Patent by European Union. http://www.prnewswire.com/news-releases/dipexium-pharmaceuticals-announces-issuance-of-locilex-patent-by-european-union-300323725.html.

[56] Newswire P. Dipexium Pharmaceuticals Announces Issuance of Locilex^®^ Patent in Japan. http://www.prnewswire.com/news-releases/dipexium-pharmaceuticals-announces-issuance-of-locilex-patent-in-japan-300241111.html.

[57] Newswire P. Dipexium Announces Top-Line Data from Onestep Phase 3 Trials with Locilex^®^ in Mild Diabetic Foot Infection Did not Meet Primary Clinical Endpoint of Superiority Versus Vehicle Plus Standardized Wound Care. http://www.prnewswire.com/news-releases/dipexium-announces-top-line-data-from-onestep-phase-3-trials-with-locilex-in-mild-diabetic-foot-infection-did-not-meet-primary-clinical-endpoint-of-superiority-versus-vehicle-plus-standardized-wound-care-300350302.html.

[58] Ladram A., Nicolas P. (2016). Antimicrobial peptides from frog skin: Biodiversity and therapeutic promises. Front. Biosci..

[59] Mu L., Tang J., Liu H., Shen C., Rong M., Zhang Z., Lai R. (2014). A potential wound-healing-promoting peptide from salamander skin. FASEB J..

[60] Chung E.M.C., Dean S.N., Propst C.N., Bishop B.M., van Hoek M.L. (2017). Komodo dragon-inspired peptide DRGN-1 promotes wound-healing of a mixed-biofilm infected wound. NPJ Biofilms Microb..

[61] Kwon Y.W., Heo S.C., Lee T.W., Park G.T., Yoon J.W., Jang I.H., Kim S.C., Ko H.C., Ryu Y., Kang H. (2017). *N*-acetylated proline-glycine-proline accelerates cutaneous wound healing and neovascularization by human endothelial progenitor cells. Sci. Rep..

[62] Pickart L., Vasquez-Soltero J.M., Margolina A. (2015). GHK peptide as a natural modulator of multiple cellular pathways in skin regeneration. Biomed. Res. Int..

[63] Demidova-Rice T.N., Geevarghese A., Herman I.M. (2011). Bioactive peptides derived from vascular endothelial cell extracellular matrices promote microvascular morphogenesis and wound healing in vitro. Wound Repair Regen..

[64] Banerjee P., Suguna L., Shanthi C. (2015). Wound healing activity of a collagen-derived cryptic peptide. Amino Acids.

[65] Tang J., Liu H., Gao C., Mu L., Yang S., Rong M., Zhang Z., Liu J., Ding Q., Lai R. (2014). A small peptide with potential ability to promote wound healing. PLoS ONE.

[66] Fife C., Mader J.T., Stone J., Brill L., Satterfield K., Norfleet A., Zwernemann A., Ryaby J.T., Carney D.H. (2007). Thrombin peptide chrysalin^®^ stimulates healing of diabetic foot ulcers in a placebo-controlled phase i/ii study. Wound Repair Regen..

[67] Sheets A.R., Demidova-Rice T.N., Shi L., Ronfard V., Grover K.V., Herman I.M. (2016). Identification and characterization of novel matrix-derived bioactive peptides: A role for collagenase from santyl(r) ointment in post-debridement wound healing?. PLoS ONE.

[68] Demidova-Rice T.N., Wolf L., Deckenback J., Hamblin M.R., Herman I.M. (2012). Human platelet-rich plasma- and extracellular matrix-derived peptides promote impaired cutaneous wound healing in vivo. PLoS ONE.

[69] Kwon Y.W., Heo S.C., Jang I.H., Jeong G.O., Yoon J.W., Mun J.H., Kim J.H. (2015). Stimulation of cutaneous wound healing by an fpr2-specific peptide agonist wkymvm. Wound Repair Regen..

[70] Sheets A.R., Massey C.J., Cronk S.M., Iafrati M.D., Herman I.M. (2016). Matrix- and plasma-derived peptides promote tissue-specific injury responses and wound healing in diabetic swine. J. Transl. Med..

[71] Maquart F.X., Pasco S., Ramont L., Hornebeck W., Monboisse J.C. (2004). An introduction to matrikines: Extracellular matrix-derived peptides which regulate cell activity. Implication in tumor invasion. Crit. Rev. Oncol. Hematol..

[72] Arul V., Gopinath D., Gomathi K., Jayakumar R. (2005). Biotinylated ghk peptide incorporated collagenous matrix: A novel biomaterial for dermal wound healing in rats. J. Biomed. Mater. Res. B Appl. Biomater..

[73] Arul V., Kartha R., Jayakumar R. (2007). A therapeutic approach for diabetic wound healing using biotinylated ghk incorporated collagen matrices. Life Sci..

[74] Loo Y., Goktas M., Tekinay A.B., Guler M.O., Hauser C.A., Mitraki A. (2015). Self-assembled proteins and peptides as scaffolds for tissue regeneration. Adv. Healthc. Mater..

[75] Rajangam K., Behanna H.A., Hui M.J., Han X., Hulvat J.F., Lomasney J.W., Stupp S.I. (2006). Heparin binding nanostructures to promote growth of blood vessels. Nano Lett..

[76] Mammadov R., Mammadov B., Toksoz S., Aydin B., Yagci R., Tekinay A.B., Guler M.O. (2011). Heparin mimetic peptide nanofibers promote angiogenesis. Biomacromolecules.

[77] Senturk B., Mercan S., Delibasi T., Guler M.O., Tekinay A.B. (2016). Angiogenic peptide nanofibers improve wound healing in stz-induced diabetic rats. ACS Biomater. Sci. Eng..

[78] Han G., Ceilley R. (2017). Chronic wound healing: A review of current management and treatments. Adv. Ther..

[79] Chattopadhyay S., Raines R.T. (2014). Review collagen-based biomaterials for wound healing. Biopolymers.

[80] Vigneswaran Y., Han H., De Loera R., Wen Y., Zhang X., Sun T., Mora-Solano C., Collier J.H. (2016). Peptide biomaterials raising adaptive immune responses in wound healing contexts. J. Biomed. Mater. Res. Part A.

[81] Cereceres S., Touchet T., Browning M.B., Smith C., Rivera J., Höök M., Whitfield-Cargile C., Russell B., Cosgriff-Hernandez E. (2015). Chronic wound dressings based on collagen-mimetic proteins. Adv. Wound Care.

[82] Tallis A., Motley T.A., Wunderlich R.P., Dickerson J.E., Waycaster C., Slade H.B., Collagenase Diabetic Foot Ulcer Study Group (2013). Clinical and economic assessment of diabetic foot ulcer debridement with collagenase: Results of a randomized controlled study. Clin. Ther..

[83] Choudary D., Insen S.G., Goyal S., Chabbra U., Singal G. (2017). A comparative study of collagen dressings versus conventional dressings in wound healing in chronic ulcers. J. Evol. Med. Dent. Sci..

[84] Ahmed S., Ikram S. (2016). Chitosan based scaffolds and their applications in wound healing. Achiev. Life Sci..

[85] Chen S., Zhang M., Shao X., Wang X., Zhang L., Xu P., Zhong W., Zhang L., Xing M., Zhang L. (2015). A laminin mimetic peptide sikvav-conjugated chitosan hydrogel promoting wound healing by enhancing angiogenesis, re-epithelialization and collagen deposition. J. Mater. Chem. B.

[86] Silva J.P., Dhall S., Garcia M., Chan A., Costa C., Gama M., Martins-Green M. (2015). Improved burn wound healing by the antimicrobial peptide llkkk18 released from conjugates with dextrin embedded in a carbopol gel. Acta Biomater..

[87] Xiao Y., Reis L.A., Feric N., Knee E.J., Gu J., Cao S., Laschinger C., Londono C., Antolovich J., McGuigan A.P. (2016). Diabetic wound regeneration using peptide-modified hydrogels to target re-epithelialization. Proc. Natl. Acad. Sci. USA.

[88] Grek C.L., Prasad G.M., Viswanathan V., Armstrong D.G., Gourdie R.G., Ghatnekar G.S. (2015). Topical administration of a connexin43-based peptide augments healing of chronic neuropathic diabetic foot ulcers: A multicenter, randomized trial. Wound Repair Regen..

[89] National Library of Medicine. A Study of Granexin Gel in the Treatment of Diabetic Foot Ulcer (Identifier: Nct02667327). https://clinicaltrials.gov/ct2/show/NCT02667327.

[90] Seo E., Lim J.S., Jun J.B., Choi W., Hong I.S., Jun H.S. (2017). Exendin-4 in combination with adipose-derived stem cells promotes angiogenesis and improves diabetic wound healing. J. Transl. Med..

